# The preterm heart: a unique cardiomyopathy?

**DOI:** 10.1038/s41390-019-0301-3

**Published:** 2019-01-19

**Authors:** Adam J. Lewandowski

**Affiliations:** Oxford Cardiovascular Clinical Research Facility, Division of Cardiovascular Medicine, Radcliffe Department of Medicine, University of Oxford, Oxford, UK

Preterm birth affects ~10% of births worldwide. Due to current advances in perinatal clinical care, survival rates >90% are regularly achieved for preterm neonates, meaning the population of adults born preterm has risen sharply in recent decades.^1^ Consequently, the first opportunities to assess longer-term disease risk of modern cohorts of smaller, more developmentally immature preterm-born survivors are only just emerging. Epidemiological studies have now identified preterm birth as an independent risk factor for cardiovascular disease, including hypertension, atrial fibrillation, stroke, and early heart failure.^2^ This increased risk is believed to be partly due to disrupted organ development as a result of an early transition from a lower resistance placental circulation during foetal development to a higher resistance arterial circulation postnatally, which is exacerbated by preterm-related complications. In line with an increased cardiovascular risk, studies using cardiovascular magnetic resonance (CMR) imaging in young adults born preterm were the first to show that they have potentially adverse alterations in both left ventricular (LV) and right ventricular (RV) structure and function.^1,3^ More recent studies have demonstrated that the early postnatal period may be a key developmental window during which these cardiac geometric and functional changes first emerge.^4^

In this issue of *Pediatric Research*, Cox et al.^5^ provide further, detailed insight into the remodelling pattern of the preterm heart during the critical postnatal window. Thirty-four preterm infants underwent CMR scans of the LV and RV within the first week following delivery (postnatal age 3–7 days) and 29 were scanned again at term-corrected age (postnatal age 33–136 days; 37–42 weeks’ corrected gestational age). Ten term-born controls underwent the same CMR scan protocol at a single time point (postnatal age 2–7 days). The authors showed that preterm-born individuals had significantly elevated weight-indexed LV mass and LV end-diastolic volume (EDV) at term-corrected age. Furthermore, weight-indexed RV mass and RVEDV trended towards being greater in the preterm group at term-corrected age, which reached statistical significance in the cohort born at 29–32 weeks’ gestational age. Cox et al. used the CMR scans in order to create computational atlases of the LV and RV from end-diastolic phases, demonstrating that preterm hearts have a more globular LV shape with more spherical blood pool. Interestingly, the degree of prematurity, requirement for respiratory support >48 h and the administration of antenatal glucocorticoids were all independently correlated with increased LV wall thickness in the preterm-born infants.

In humans, the heart undergoes substantial remodelling over the first days and weeks of postnatal life, with the RV switching from being a thick-walled chamber that provides two-thirds of cardiac output into the systemic circulation to being the relatively thinner walled, crescent-shaped chamber that supplies the lower pressure pulmonary circulation.^3^ Although follow-up of the preterm cohort in the study by Cox et al. was at term-corrected age (37–42 weeks’ corrected gestational age), there was large variation in postnatal age for their second scans (33–136 days). We and others have shown that the LV and RV in both preterm-born and term-born individuals undergoes extensive cardiac remodelling postnatally,^4^ thus additional follow-up in the term-born group after several weeks of exposure to the extrauterine environment using the same methodology would be required to fully understand differences in physiological adaptation related to birth gestational age. Nevertheless, Cox et al.’s findings related to ventricular volumes in the preterm group at term-corrected age may be a reflection of the further enhanced physiological adaptation to increased pulmonary venous return in those born at earlier gestations.^4,5^ Given that LV and RV volumes are reduced in those born preterm in childhood and young adulthood,^1,3,6^ the current study highlights the need for longitudinal cardiac imaging studies tracking the same individuals over time to better understand the evolution of these remodelling patterns throughout development.

Cardiac changes in preterm-born individuals are of clinical concern. In longitudinal studies, the 20% increase in LV mass seen in young adults born moderately preterm based on CMR imaging is equivalent to >50% increased risk of cardiovascular clinical events in later adult life.^1^ The 64% increase in LV mass seen in those born at less than 29 weeks’ gestation in the study by Cox et al. is therefore of particular concern if these changes track throughout development. The nature of this myocardial thickening remains to be determined, but animal studies of preterm suggest that both cardiomyocyte hypertrophy and fibrosis are early pathophysiological adaptations,^7^ even in the absence of the normal inflammatory and stress signals present in human preterm pregnancies. Studies have also identified that RV systolic function is reduced from early in life,^4^ with clinically significant reductions in young adulthood that may directly contribute to the onset of clinical heart failure.^3^ Indeed, Carr et al. have demonstrated in a large Swedish register-based epidemiological study of 2.67 million individuals that those born preterm are at increased risk of incident heart failure from childhood through to young adulthood, with a fourfold increased risk in those born at 28–31 weeks’ gestation (very preterm) and 17-fold increased risk in those born at <28 weeks’ gestation (extremely preterm).^2^ Given the absolute number of incident heart failures was still greater in those born at term, this may be due to the altered myocardial development in those born preterm, as a reduced myocardial reserve would make them more susceptible to acute insults commonly causing early heart failure. In accordance with this hypothesis, we recently demonstrated using echocardiography imaging at prescribed exercise intensities that preterm-born young adults have impaired LV functional response to physical exercise.^8^ Additionally, using right heart catheterisation, Goss et al.^9^ demonstrated that young adults born preterm were significantly less able to augment cardiac index or right ventricular stroke work during exercise. Despite the independence of the changes in LV and RV structure and function from blood pressure, exposure to the known sustained blood pressure elevation, hypertension and other cardiovascular risk factors in preterm-born individuals might have a greater impact over time in these individuals due to this abnormal pattern of cardiac remodelling and reduced myocardial reserve.

The findings from Cox et al. and others have demonstrated the importance of this early postnatal window for growth, development and cardiac remodelling in preterm infants. As such, it may be an ideal period for intervention to prevent future risk of cardiovascular disease. In a hyperoxia-exposed rat model mimicking preterm birth-related stress conditions, early treatment with an angiotensin II type 1 (AT1) receptor antagonist, Losartan, prevented the development of cardiac alterations in later life, including fibrosis and hypertrophy.^7^ These findings suggest that intervention in the first days and weeks postnatally can alter the long-term course of cardiac disease risk. Though humans are more likely to be faced with a greater number of confounding variables and environmental factors affecting development and risk throughout life, our previous work has shown the possible benefits of intervention during the early postnatal window using a more practical approach for human infants.^10^ By performing a follow-up study in a cohort of preterm-born young adults who had been randomised to different milk feeding diets at birth between 1982–1985, we were able to investigate the potential long-term cardiac remodelling benefits of an exclusive human milk diet in immediate preterm postnatal life. We performed detailed cardiac phenotyping using CMR imaging and computational cardiac atlas formation to explore cardiac remodelling patterns in young adulthood. Comparison of young adults who were fed exclusively human milk versus those who were fed exclusively on formula as infants revealed that the LV and RV end-diastolic and stroke volumes in the group fed exclusively human milk approached values seen in term-born controls, with particularly striking findings for the RV. The findings implicate early preterm postnatal life as a potentially tractable period of cardiovascular development, relevant to long-term outcomes, and support promotion of human milk for the care of preterm infants to reduce long-term cardiovascular risk. Future work is needed to understand potential benefits of different variations of supplemental feeding and fortifiers to support normal growth and development in very and extremely preterm-born neonates. Furthermore, whether other interventions during the perinatal period or throughout life, such as pharmacological and prescribed dietary and exercise advice, can help preferentially reduce adverse cardiac remodelling in preterm-born individuals remains to be determined but should be a primary focus of current research in the field.

In conclusion, Cox et al. provide further evidence of a unique cardiac phenotype in offspring born preterm and should be commended for their sophisticated methodological approach using CMR and cardiac atlas formation in neonates and infants to better define these patterns of geometric remodelling. The increasing body of evidence from animal models and humans born preterm demonstrating a unique cardiac morphology and abnormal functional stress response provides mechanistic insight to the findings from epidemiological studies. On the whole, this supports the notion that being born preterm is associated with a unique cardiomyopathy. Understanding which individuals born preterm are at greatest risk and what leads to the heterogeneity in the preterm cardiac phenotype remains to be further explored, but immediate consideration for long-term clinical cardiovascular follow-up in preterm-born individuals is warranted.
